# Recent Advances on Cell-Based Co-Culture Strategies for Prevascularization in Tissue Engineering

**DOI:** 10.3389/fbioe.2021.745314

**Published:** 2021-11-25

**Authors:** Sepehr Shafiee, Siavash Shariatzadeh, Ali Zafari, Alireza Majd, Hassan Niknejad

**Affiliations:** Department of Pharmacology, School of Medicine, Shahid Beheshti University of Medical Sciences, Tehran, Iran

**Keywords:** co-culture, pre-vascularization, tissue engineering, endothelial cells, mesenchymal stem cells, fibroblast, perivascular cells, endothelial progenitor cells

## Abstract

Currently, the fabrication of a functional vascular network to maintain the viability of engineered tissues is a major bottleneck in the way of developing a more advanced engineered construct. Inspired by vasculogenesis during the embryonic period, the *in vitro* prevascularization strategies have focused on optimizing communications and interactions of cells, biomaterial and culture conditions to develop a capillary-like network to tackle the aforementioned issue. Many of these studies employ a combination of endothelial lineage cells and supporting cells such as mesenchymal stem cells, fibroblasts, and perivascular cells to create a lumenized endothelial network. These supporting cells are necessary for the stabilization of the newly developed endothelial network. Moreover, to optimize endothelial network development without impairing biomechanical properties of scaffolds or differentiation of target tissue cells, several other factors, including target tissue, endothelial cell origins, the choice of supporting cell, culture condition, incorporated pro-angiogenic factors, and choice of biomaterial must be taken into account. The prevascularization method can also influence the endothelial lineage cell/supporting cell co-culture system to vascularize the bioengineered constructs. This review aims to investigate the recent advances on standard cells used in in vitro prevascularization methods, their co-culture systems, and conditions in which they form an organized and functional vascular network.

## 1 Introduction

**FIGURE 1:**
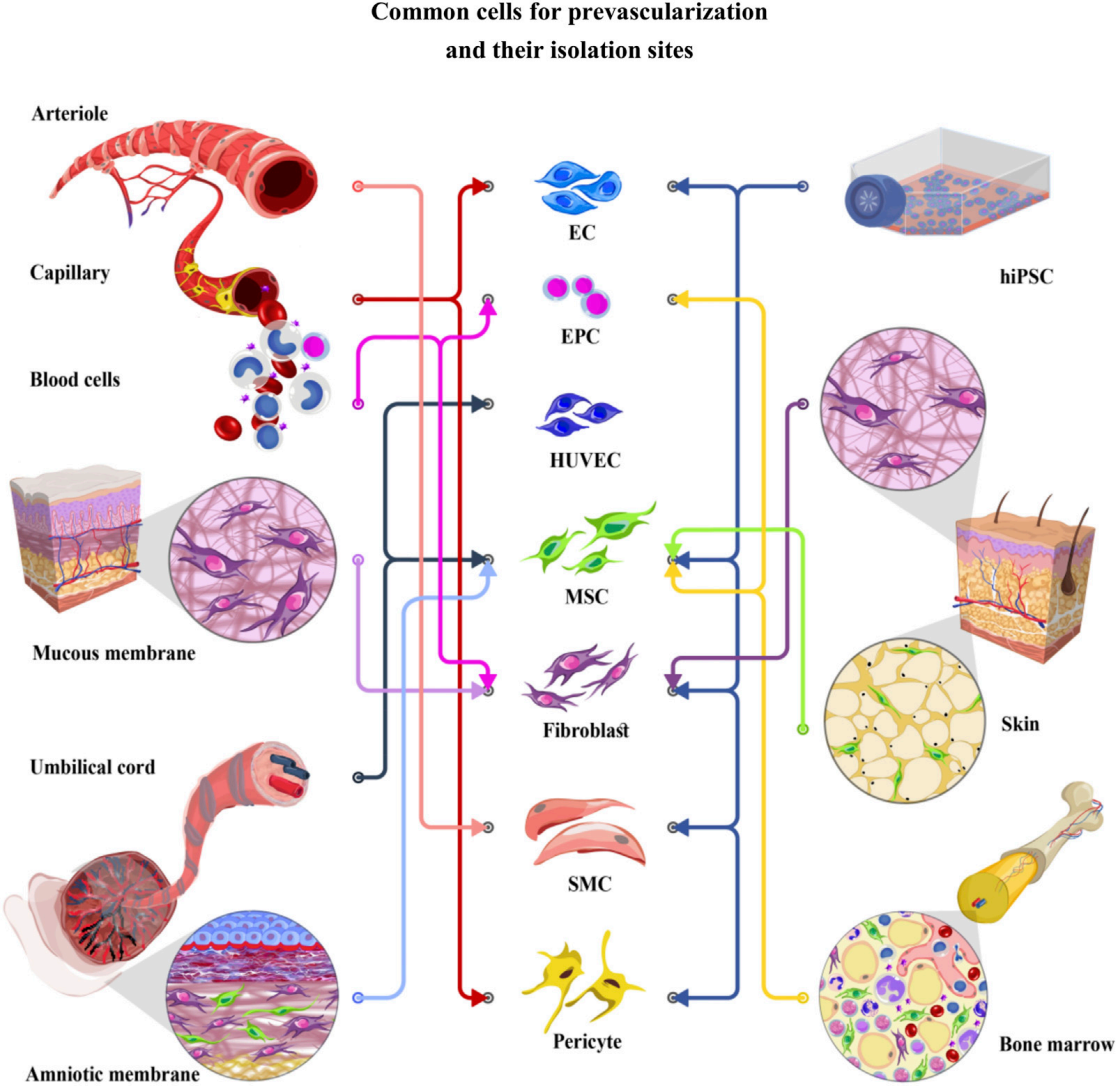
Common cells for prevascularization and their isolation sites. EC (Endothelial cells), EPC (Endothelial progenitor cells), HUVEC (Human umbilical vein endothelial cells), MSC (Mesenchymal stem cells), fibroblast, SMC (Smooth muscle cells), and pericyte have been used for prevascularization of different tissues. Endothelial and supporting cells can be obtained from various cell sources, including skin, mucosal membrane, bone, and peripheral blood, or differentiated from hiPSCs (human induced pluripotent stem cells). Recently, particular attention has been given to the umbilical cord and amniotic membrane as easy to access cell sources. These cells show lower immunogenicity than the other cell lineages, introducing them as excellent candidates in the prevascularization process.

The concept of tissue engineering and regenerative medicine originates from the idea of replacing damaged or dysfunctional organs with new regenerated ones. As the tissue grows, oxygen and nutrient supply as well as wastes elimination cannot be achieved by simple diffusion. Therefore, vascular or vascular-like networks are crucial for proper function and survival of any tissue. Central necrosis will happen in engineered tissues thicker than 100–250 µm if there is no efficient vascular bed (Fu et al., 2021). Therefore, developing approaches to form adequate and functional vasculature within artificial tissues and organs and prevascularization of engineered constructs prior to implantation are considered as a promising concept in tissue engineering field (Yazdanpanah et al., 2015). The survival of incorporated stem cells in scaffolds depends on the efficiency and efficacy of networks developed by prevascularization. In order to mimic the physiological structure of the capillaries, some efforts have been dedicated to design perfusable micro-channels in scaffolds (Farhadihosseinabadi et al., 2018; Figueiredo et al., 2020; Liu et al., 2020). Many studies have tried to use the physiological capability of stem cells for developing a vascular network *de novo*, known as vasculogenesis which can be usually seen in the embryonic period. To induce vasculogenesis, many *in vitro* strategies such as cell sheet engineering, cell spheroid and cell encapsulation, bio-printing and micro-fluid techniques have been introduced. The other methods of prevascularization take advantage of physiological process of angiogenesis, when new blood vessels are developed from the existing vessels (Farhadihosseinabadi et al., 2018). To date, several *in vitro*, *in vivo* and *in situ* prevascularization strategies have been employed to compose functional engineered tissues. *In vivo* prevascularization techniques such as prevascularization via AV-loop, subcutaneous implantation and flaps mostly recruit the angiogenesis process, counting on the ability of host vessels to invade the implanted scaffold (Kiaie et al., 2020; Vidal et al., 2020; Redenski et al., 2021). *In situ* methods employ a combination of *in vitro* and *in vivo* approaches. In all of these methods, endothelial lineage cells are the key part of developing a proper vascular network. Moreover, cell-to-cell interactions, biomaterials, and growth factors profoundly influence *in vitro* prevascularization. Although formation of capillary-like networks can be initiated by endothelial lineage cells, interactions of endothelial and supporting cells are essential for developing a functional vascular network (Figure 1).

## 2 Endothelial Lineage Cells

Producing a lumenized endothelial cell network is one of the main milestones of prevascularization (Kiaie et al., 2020; Später et al., 2020). Endothelial cells (ECs) which form the interior lining of blood vessels, can be obtained from various tissues including umbilical vein, aorta and different micro vessels such as micro vessels in adipose tissue or foreskin (Krüger-Genge et al., 2019). These cells can be differentiated from human induced pluripotent stem cells (hiPSCs) and pluripotent stem cells. (Natividad-Diaz et al., 2019) Furthermore, endothelial progenitor cells (EPCs) can be isolated from peripheral blood and bone marrow (Figure 1) (Sun et al., 2020). Appropriate ECs should be selected based on its isolation process and angiogenic properties. ECs are mainly identified by expressing cluster of differentiation (CD) 31 and von Willebrand factor (vWf) in co-cultures, while they express a variety of biomarkers including, Fli-1 (friend leukemia integration-1), CD13, CD29, CD36, CD34, CD39, CD44, CD47, ICAM-1 (intercellular adhesion molecule-1), CD61, CD62, CD80, CD102, CD105, CD106, CXCL16, CD143, CD144, CD146, ADAMTS13 (a disintegrin and metalloproteinase with thrombospondin motifs-13), ADAMTS18, and VE-cadherin (vascular endothelial cadherin). However, it must be taken into account that the ratio of expressed markers can be different in specialized endothelial cell networks (Goncharov et al., 2020). In recent years, different sources of endothelial cells have been used alone or in a co-culture system to form capillary network *in vitro*. In this section, we discuss the most applicable endothelial lineage cells used in prevascularization.

### 2.1 Human Umbilical Vein Endothelial Cells

Human umbilical vein endothelial cells (HUVECs), well-known for their angiogenic features, are harvested from the endothelium of the umbilical cord veins. Their angiogenic capabilities could be attributed to the existence of a progenitor cell subpopulation within HUVECs (Ingram et al., 2005; Kocherova et al., 2019). These cells express many common EC markers such as CD31, vWf, CD34, CD54, ICAM-1, CD62-E, CD106, VCAM-1 (vascular cell adhesion protein-1), and CD143 (Ma et al., 2014a; Kuss et al., 2018). HUVECs have been used in various prevascularization strategies such as bioprinting. For instance, HUVEC spheroids encapsulated in fibrin and collagen were printed with high rate of viability on scaffolds with tuned surface topological features to control the alignment of the developed capillary-bed (Benning et al., 2018). Considering their simple and low-cost isolation methods, they have been widely used in co-culture systems with a wide variety of supporting and target tissue cells. HUVECs can be co-cultured with mesenchymal stem cells (MSCs) derived from different origins. Adipose tissue mesenchymal stem cells (AD-MSCs) and bone marrow mesenchymal stem cells (BM-MSCs) are known as commonly used MSCs that are frequently co-cultured with HUVECs for prevascularization of various target tissues. BM-MSCs and AD-MSCs show similar vasculogenic capabilities. However, the isolation process of AD-MSCs is more accessible than BM-MSCs (Ma et al., 2014b). In addition to AD-MSCs and BM-MSCs, HUVECs co-culture with amniotic membrane derived mesenchymal cells have shown successful results with a ratio of 1:1 for developing prevascularized micro-tissues (Zhang S. et al., 2017).

Different factors influence the prevascularization of engineered tissues via HUVECs/MSCs co-culture systems including seeding density ratio, growth media and culture conditions. The optimal HUVECs/MSCs ratio recommended by some studies is 5:1 (Kaully et al., 2009; Rao et al., 2012; Ma et al., 2014a). Au et al. reporeted that vascular networks created by cell suspension of HUVECs with the HUVECs/BM-MSCs ratio of 5:1 in a collagen/fibronectin hydrogel remained functional for 130 days after implantation *in vivo* (Au et al., 2008).

Different studies focusing on angiogenesis within bone regeneration have used HUVECs and MSCs co-culture systems. HUVECs/MSCs co-culture can reduce mineralization of the engineered bone tissues. In this matter, subsequent seeding of endothelial cells after reaching proper confluency of MSCs can be a potent solution (Kim et al., 2019). MSCs from different origins support both network formation and osteogenic differentiation of engineered bone constructs. Human umbilical cord mesenchymal stem cells, BM-MSCs, hiPSCs derived mesenchymal stem cells (hiPSC-MSCs) and embryonic stem cell differentiated mesenchymal cells in separate cultures with HUVECs developed vessel-like network on a calcium phosphate cement (CPC) scaffold. In that study, no significant difference in capillary development and mineralization was detected between different type of MSCs which were co-cultured with HUVECs (Chen et al., 2018). Moreover, HUVECs co-cultured with hiPSC-MSCs developed capillary-like structures and promoted mineralization on CPC scaffold, so that a complex and well-organized capillary network was successfully formed at 21 days post-culture. (Liu et al., 2017).

HUVEC/BM-MSC spheroids encapsulated within collagen/fibrin hydrogels showed more favorable capillary-like structure formation and osteogenic differentiation than the cell suspension form (Heo et al., 2019). HUVEC/AD-MSC spheroids with 1:1 ratio encapsulated in hyaluronic acid/gelatin bioactive hydrogels were successfully used for prevascularizing a 3D-bioprinted Polycaprolactone/Hydroxyapatite scaffold. A significant cell migration and sprouting was observed in the co-culture system after 7 days (Kuss et al., 2018). HUVEC/AD-MSC spheroids with a ratio of 1:9 have been bio-printed to make a prevascularized adipose micro-tissue. The cell spheroids formed capillary-like structure within 7–14 days (Benmeridja et al., 2020). HUVEC/BM-MSC spheroids can develop a capillary-like network in fibrin scaffold. The optimal vessel development was achieved in the HUVEC/MSC spheroids with a 1:1 ratio. It is worth noting that there may be less chance of capillary-like developments in HUVEC spheroids alone. Furthermore, MSCs migration always happens after HUVECs migration (Roux et al., 2018)

Different models of cell sheet engineering have been applied for developing prevascularized structures in bone tissue engineering. Zhang et al. developed a prevascularized natural nanofibrous extracellular matrix sheet with high mechanical strength using HUVECs/MSCs (Zhang et al., 2018). Likewise, HUVECs/AD-MSCs magnetic responsive sheets have been used for developing an engineered bone tissue by internalizing iron oxide nanoparticles in the cells (Silva et al., 2020). Furthermore, osseous cell sheets created by osteo-induction of AD-MSCs have been applied as a bio-paper for bio-printing HUVECs via laser-assisted bioprinting (Kawecki et al., 2018).

Dynamic co-culture and physical tension could enhance both angiogenic and osteogenic differentiation. Culturing HUVECs with BM-MSCs within a dynamic condition of tubular perfusion system bioreactor showed an increase in production of bone morphogenic protein 2 (BMP-2) and development of CD31 network indicating osteogenesis and vasculogenesis (Nguyen et al., 2017). It was concluded that tension could promote osteogenic properties of HUVECs/BM-MSCs cultures (Jiang et al., 2018).

HUVECs/MSCs have been also used for developing a prevascularized engineered cardiac constructs. In a study conducted by Sharma et al. a confluent culture of MSCs on a fibroblast-derived nanoscale extracellular matrix (ECM) scaffold formed a CD166 tracks aligned with ECM nano-fibers. Subsequent seeding of HUVECs on the developed scaffold resulted in formation of a well-organized capillaries aligned with CD166 tracks (Qian et al., 2019). Culturing HUVECs and fibroblasts on a micro-patterned polyethersulfone/polyvinylpyrrolidone membranes also formed an aligned network with topological features of membranes (Skrzypek et al., 2018). It seems that the origin of MSCs profoundly affect the vascularization capability of HUVECs. HUVECs in culture with dermal fibroblast, AD-MSCs, BM-MSCs and Wharton’s jelly-MSCs were used to prevascularize both agarose-collagen and fibrin hydrogels. Particularly, capillary networks were more noteworthy in co-cultures on fibrin hydrogels as well as HUVECs/fibroblasts and AD-MSCs co-culture, while co-cultures containing BM-MSCs developed less significant capillary-like structures (Kniebs et al., 2020).

### 2.2 Endothelial Progenitor Cells

Endothelial Progenitor Cells (EPCs), which are circulating in the peripheral blood, play a crucial part in regeneration of vasculature bed endothelial lining. EPCs are also more proliferative and potent than the adult endothelial cells, where their pro-angiogenic properties are more noteworthy than that of non-progenitor endothelial cells (Asahara et al., 1997; Aicher et al., 2006). They can be derived from bone marrow, peripheral blood, umbilical cord blood and also can be harvested by differentiation of the hiPSCs (Garikipati and Kishore, 2017; Huizer et al., 2017). Endothelial colony-forming cells (ECFCs) are adult EPCs that take part in vasculogenesis and regenerating endothelial lining. The EPCs derived from human cord blood (human cord blood endothelial colony-forming cells or hCB-ECFCs) show much greater proliferative and angiogenic abilities compared with EPCs isolated from human peripheral blood (hPB-ECFCs) (Peters, 2018). Until now, EPCs have been used for vascularizing common scaffolds such as decellularized scaffolds (Guo et al., 2018), Poly-Ethylene Glycol Hydrogel (Peters et al., 2016), and Matrigel (Iqbal et al., 2017; Rosca et al., 2018). With all the controversies over the EPCs and their applications, they show promising potentials for scaffold vascularization (Peters, 2018). EPCs ability to develop capillary-like networks in culture with osteoblasts (Fuchs et al., 2007), fibroblasts (Dai et al., 2018) and mesenchymal stem cells (Liu H. et al., 2018) has been demonstrated in several studies.

In spite of many encouraging results obtained from using EPCs for prevascularization, some concerns still remain regarding their origin and classification. In fact, there is variation in definition of EPCs phenotype and their distinctive features in the literature (Table 1). Some authors divided EPCs into two categories, including hematopoietic EPCs which are stem cells originated from bone marrow with pro-vasculogenic properties, and non-hematopoietic EPCs which have ECs phenotype with less potency for pro-vasculogenic features (Garikipati and Kishore, 2017). In another classification, these cells have been categorized into two subpopulations including early EPCs and late EPCs with different angiogenic features. Early EPCs are spindle shaped and possess extensive proliferative abilities in the second and third week after isolation; however, their proliferative potential is limited after 6 weeks. It has been reported that they are also involved in innate immunity and inflammation. As a result, their application is limited because they may raise inflammatory responses and consequently cause graft rejection (Cheng et al., 2013). Late EPCs are cobblestone shaped that form primitive colonies after 3 weeks and are far more proliferative than early EPCs (Fedorovich et al., 2010). There is an agreement now that late EPCs contribute in angiogenesis and the early EPCs may indirectly join in endothelial network formation (Medina et al., 2010). Although there are many controversies surrounding the specific biomarkers for EPCs isolation, some markers such as CD34, CD133, and VEGFR2 (vascular endothelial growth factor receptor-2) have been frequently used for identification of these cells (Sen et al., 2011).

**TABLE 1:** Common markers used for isolation of endothelial cells and supporting cells.

Cell type	Specific cell category		Markers used for isolation		References
			Positive	Negative	
Endothelial lineage cells	HUVECs		CD31, CD34, CD40, CD54, CD62-E, CD106, CD143, vWf, ICAM-1, VCAM-1, VE-cadherin, ADAMTS13, ADAMTS18	-	Chopra et al. (2018)
	EPCs				Bouïs et al. (2001)
		*Early EPCs*	CD14, CD31, CD45, CD133, vWF	-	Peters (2018)
		*Late EPCs(OECs)*	CD31, CD34, CD133, vWF, VEGFR-2, VE-cadherin	CD14, CD45, CD115	Jang et al. (2019)
		*Non-hematopoietic EPCs*	CD31, CD34, CD105, CD146, VEGFR-2, VE-cadherin	CD133	Hofmann et al. (2009)
		*Hematopoietic EPCs*	CD31, CD34, CD105, CD133, VEGFR-2, CXCR-4, c-Kit	-	Garikipati and Kishore, (2017)
		*ECFCs*	CD31, CD34, CD44, vWF, VEGFR-2, VE-cadherin	CD14, CD45, CD115, CD133	Khaki et al. (2018)
		*CECs*	CD31, CD34, CD44, CD146, vWF, VEGFR-2, VE-cadherin	CD45	Oswald et al. (2004)
	Microvascular and differentiated endothelial cells				Müller et al. (2002)
		*hDMECs*	CD31, CD36, CD40, CD144, vWF	-	Mukai et al. (2008)
		*hiPSC-ECs*	CD31, CD49d, CD105, CD144, vWF, VEGFR-2, VE-cadherin	-	Sukmawati and Tanaka, (2015)
		*MSC-ECs*	CD31, CD44, CD73, CD105, CD144, vWF, VCAM-1, FLT-1, VEGFR-2, VE-cadherin	-	Swerlick et al. (1992)
					Xing et al. (2020)
Supporting cells	MSCs		CD11, CD44, CD73, CD90, CD105, CD106, CD166, Integrin-α1, IGF-2	CD11b, CD19, CD34, CD45, HLA-DR	De Souza et al. (2016a)
	Fibroblasts		CD9, CD29, CD44, CD73, CD90, CD105, CD166, MMP-1, MMP-3	CD146	Lynch and Watt, (2018)
	Pericytes		CD73, CD90, CD105, CD146, PDGFRβ, α-SMA, NG2, Desmin, RGS5	-	Owens and Wise (1997), Xueyong et al. (2008)
	VSMCs		CD73, CD90, CD105, α-SMA, PDGFRβ, H-caldesmon, Smoothelin, Calponin	-	Viswanathan et al. (2019)

HUVECs, Human umbilical vein endothelial cells; EPCs, Endothelial progenitor cells; OECs, Outgrowth endothelial cells; ECFCs, Endothelial colony forming cells; CECs, Circulating endothelial cells; hDMECs, Human dermal microvascular endothelial cells; hiPSC-ECs, Human induced pluripotent stem cells derived endothelial cells; MSC-EC, Mesenchymal stem cell-derived endothelial cells; MSCs, Mesenchymal stem cells; VSMCs, Vascular smooth muscle cells; CD, Cluster of differentiation; vWF, von Willebrand factor; ICAM-1, Intercellular adhesion molecule-1; VCAM-1, Vascular cell adhesion molecule-1; ADAMTS, A disintegrin and metalloproteinase with thrombospondin motifs;VEGFR-2, Vascular endothelial growth factor receptor 2;VE-cadherin, Vascular endothelial cadherin;CXCR-4, C-X-C chemokine receptor type 4; FLT-1, FMS-like tyrosine kinase-1; IGF-2, Insulin-like growth factor-2; HLA-DR, Human leukocyte antigen-DR; MMP, Matrix metalloproteinase; PDGFRβ, Platelet-derived growth factor receptor β; α–SMA, α-Smooth muscle actin; NG-2, Neuron-glial antigen 2; RGS-5, Regulator of G-protein signaling 5.

Recently, EPCs co-cultured with different supporting cells have been successfully used for prevascularization. Growth factors released from MSCs, including VEGF (vascular endothelial growth factor) and bFGF (basic fibroblast growth factor), enhance angiogenic capability of EPCs (Fauza et al., 2018). EPCs from peripheral blood combined with peripheral blood MSCs were used for prevascularizing a 3D biphasic calcium phosphate bio-ceramic. In that study, the expression of VEGF, PDGF (platelet-derived growth factor) and alkaline phosphatase significantly increased in EPCs/MSCs co-culture (Chen et al., 2019). It has been reported that the optimal angiogenic and osteogenic value were observed in in PB-EPCs/BM-MSCs cultures within 1:3 or 2:1 ratio (Peng et al., 2019). Moreover, a prevascularized patch with antifibrotic properties have been developed by bioprinting EPCs and BM-MSCs on a decellularized liver scaffold (Wonil et al., 2018).

EPCs isolated from cord blood in culture with fibroblasts developed a capillary-like network in 7 days with a similar morphology to HUVECs/fibroblasts co-culture. Interestingly, EPCs/fibroblasts made anastomosis with the host vasculature earlier than HUVECs/fibroblasts and signs of blood perfusion was appeared after 1 day of implantation. It was observed that higher ratio of fibroblasts can result in stronger anastomosis to the host vasculature (Chen et al., 2010). Lee et al. used a co-culture system by peripheral blood isolated EPCs and human mucosal fibroblasts and keratinocytes to develop a skin tissue construct. They seeded EPCs on a fibroblast-incorporated fibrin layer. Then, keratinocytes were seeded subsequently on the developed scaffold. It was showed that their co-cultured system formed a prevascularized construct whit successful results in the excisional wound of a nude mice (Lee et al., 2019).

### 2.3 Microvascular and Adult Endothelial Cells

Adult endothelial cells have limited proliferation. These cells are highly specialized and exhibit unique morphology and characteristics in tissues like liver and kidney, which limit their potential application in prevascularization (Bale et al., 2015). However, microvascular endothelial cells such as human dermal microvascular endothelial cells (hDMECs), adult human cardiac endothelial cells (hCECs), adult human pulmonary artery endothelial cells (hPAECs), and aortic endothelial cells have been successfully used for prevascularization of engineered tissue constructs (Jiang et al., 2018; Manikowski et al., 2018). For instance, aortic endothelial cells in co-culture with BM-MSCs developed microvasculature after 14 days of culture (Pekozer et al., 2016).

MSCs co-culture with other adult endothelial cells such as human cardiac microvascular endothelial cells (hCMVECs) has been found to be effective to produce a functional prevascularized 3D cardiac graft (Valarmathi et al., 2018). hCMVECs in the present of MSCs could successfully form a capillary-like network in a 3D collagen cell carrier (CCC) within 7 days under vasculogenic conditions. Later, hiPSC-derived embryonic cardiomyocytes were cultured on the prevascularized CCC under myogenic conditions for another week to achieve myogenic differentiation (Valarmathi et al., 2017). Human adipose tissue microvascular endothelial cells (hAMECs) are potent candidates for prevascularization of skin and soft tissue substitutes. Promising results have been reported in co-seeding of hAMECs and adult normal human dermal fibroblasts to form a well-organized vascular network. The optimal mechanical strength and capillary development are achieved in ECs/fibroblasts ratio of 4:1. It is observed that modifying the ECs/fibroblasts ratio can change the pattern of vascular network, giving researchers more choices for prevascularization of different tissues (Czajka and Drake, 2014). For instance, human dermal microvessel endothelial cells isolated from foreskin in culture with gingival fibroblasts and gingival epithelial cells, by an ECs/fibroblasts ratio of 1:1 were used to develop a prevascularized buccal mucosa substitute, with the potential application in urethral defects (Heller et al., 2016). In another example, a complex capillary-like network was developed by human dermal fibroblasts (hDFs) co-cultured with hDMECs with ratio 1:1 in thermoresponsive biomimetic polyisocyanopeptide (PIC) with pore diameters of 100–150 μm (Zimoch et al., 2018). In another study Sasagawa et al. created a construct using human aortic endothelial cells entrapped between two hDFs cell sheets that successfully developed a CD31^+^ network within 3 days (Sasagawa et al., 2014).

### 2.4 Stem Cell-Derived Endothelial Cells

Finding an autologous endothelial cell source is one of the main challenges of prevascularization. Endothelial cells can be differentiated from different stem cell sources such as MSCs, hiPSCs, and also from embryonic stem cells, fetal pluripotent stem cells, and totipotent embryo stem cells (Xu et al., 2019). Several strategies have been employed for the endothelial differentiation of stem cells. Most of these strategies use an endothelial differentiation medium that contains growth factors such as VEGF (Oswald et al., 2004; Wu et al., 2007; Wang et al., 2018). A 2D monolayer or a 3D embryoid body of hiPSCs or embryonic stem cells can be differentiated into endothelial lineage cells using an endothelial differentiation medium (Kim et al., 2007; Zhang J. et al., 2017; Suresh and West, 2020). Furthermore, the pluripotent stem cells can be co-cultured with OP9 stromal cells that produce endothelial differentiating factors (Figueiredo et al., 2015). As another strategy, genetic modification can be used for endothelial differentiation of the embryonic stem cells and hiPSCs. For Instance, Wang et al. differentiated endothelial cells from hiPSCs by altering the transcription factor E26 transformation-specific variant 2 or ETV2 gene (Wang K. et al., 2020). Lindgren et al. also modified the expression of ETV2 for endothelial differentiation of embryonic stem cells (Lindgren et al., 2015). In addition to ETV2, alteration of the other transcriptional factors such as GATA-2, LMO-2, and TAL-1 have been proposed as a strategy for endothelial differentiation of stem cells (Elcheva et al., 2014; Lange et al., 2020). After endothelial differentiation, endothelial-specific markers such as CD31, CD34, CD144, VEGFR-2, and vWF have been used for confirming the endothelial differentiation of stem cells (Kennedy et al., 2021). There are still several questions regarding the functionality of endothelial cells differentiated from hiPSCs. Li et al. observed that endothelial cells derived from hiPSC have a significantly decreased proliferation rate than those derived from embryonic stem cells, and they can lose their endothelial phenotype and markers through passages (Li et al., 2011). Although ECs differentiated from hiPSCs have been used to develop functional capillary beds, it has been shown that the vascular network developed by hiPSCs had less density than the vascular network created by HUVECs (Bezenah et al., 2018). Further investigations are required on the effect of various endothelial differentiation strategies on network forming ability of stem cell-derived endothelial cells.

Several studies used stem cell-derived endothelial cells for prevascularization. Culture of various ECs, such as hCECs, hPAECs, hiPSCs derived endothelial cells (hiPSC-ECs) with adipose-derived stromal cells successfully developed a well-formed capillary-like network (Calderon et al., 2017; Manikowski et al., 2018). However, it was observed that without external growth factors, the developed network collapsed, and ECs mono-culture could not complete the capillary-like network (Manikowski et al., 2018). In another study, co-culture of ECs differentiated from BM-MSCs of Witsar rat with BM-MSCs have been used for developing a scaffold-free cell sheet. Accordingly, the development of a capillary-like network and lumen formation was observed within a week that eventually promoted repair of the rat cranial bone defects (Xu et al., 2019). Moreover, MSC-derived CD31^+^ endothelial cells in culture with AD-MSCs and MSC-derived fibroblasts were successfully used for prevascularizing a fibroblast niche coated tissue engineered dermal graft (TEDG) scaffold, which was developed as a skin substitute (Ajit et al., 2020). In another study, Masuda et al. developed a cardiac cell sheet using rat embryonic stem cell-derived ECs, dermal fibroblasts and embryonic stem cell-derived cardiomyocytes. It was reported that cardiomyocytes could promote endothelial cell differentiation and sprouting (Masuda et al., 2015). Overall, stem cell-differentiated endothelial cells represent a valid and promising cell source for prevascularization. However, prior to clinical application of these cells in prevascularized constructs, it is important to resolve concerns regarding the efficiency of these cells in fabricating endothelial networks, and their teratogenicity.

## 3 Supporting Cells

To achieve proper perfusion in engineered tissue constructs, the quality of vessels is as vital as their length and sprout numbers. Development of these functional networks can be achieved using supporting cells along with endothelial cells. Combination of endothelial lineage cells with target tissue cells and supporting cells like fibroblasts (Sorrell et al., 2007), smooth muscle cells (Fillinger et al., 1997), mesenchymal stem cells (Aguirre et al., 2010), pericyte cells (Antonelli-Orlidge et al., 1989), epithelial cells (Kim et al., 2002), and osteoblasts (Hofmann et al., 2008) seems to be a potential solution for vascularization of engineered tissue constructs. Supporting cells affect the formation and organization of capillary networks directly, while target tissue cells such as cardiomyocytes, osteoblasts, epithelial cells, keratinocytes, and neural cells have an indirect effect (Grellier et al., 2009). Supporting cells facilitate inosculation to the host vasculature in addition to their ability to improve ECs viability and proliferation and they also stabilize newly formed vessels by inhibiting uncontrolled angiogenesis. The effectiveness and supportive role of supporting cells are owing to the production of growth factors and ECM, resulting in stabilizing the capillary-like formations. In fact, these cells not only support and stabilize newly formed capillaries, but also act as a source of pro-angiogenic growth factors such as VEGF and bFGF.

### 3.1 Mesenchymal Stem Cells

Mesenchymal stem cells, as the first group of supporting cells, are able to differentiate into various cell types such as pericytes, adipocytes, chondrocytes, myocytes and osteoblasts (Caplan, 1991; Abbasi-Kangevari et al., 2019). They can be harvested from different tissues including bone marrow (BM-MSCs), adipose tissue (AD-MSCs), placenta, umbilical cord, amniotic fluid, peripheral blood (PB-MSC), dental pulp, limbal stroma as well as differentiated hiPSCs (Funderburgh et al., 2016). They express various surface markers including CD11, CD44, CD73, CD90, CD105, CD106, and CD166. However, these cells do not express CD14, CD34, and CD45 (Avolio et al., 2017; Valarmathi et al., 2018) (Table 1). The angiogenic ability of MSCs is differ from each other, so that they morphologically form different vessel networks depending on their origins. For example, the morphology of vessels induced by umbilical artery, umbilical vein and “Wharton’s jelly” mesenchymal cells are different (Xu et al., 2017).

AD-MSCs are preferred in many studies due to their simple isolation and their ability to induce angiogenesis (Chen et al., 2015; Freiman et al., 2016). The umbilical-derived mesenchymal cells have a great angiogenic ability, while the placenta-derived MSCs show a higher proliferation rate, multi-lineage differentiation capability and lower immunogenicity (Kargozar et al., 2018). It is worth to mention that the hiPSC-MSCs and BM-MSCs both promote vascularization, even though telomerase activity is 10-fold greater in hiPSC-MSCs than in BM-MSCs (Chen et al., 2018).

MSCs have been used in many studies due to their abilities to promote vessel formation and maturation by different mechanisms (Navone et al., 2014; Oki et al., 2018). The MSCs produce pro-angiogenic cytokines such as hypoxia-inducible factor 1α (HIF-1α), angiogenin, angiopoietin I, angiopoietin II, angiopoietin IV, interleukin-1β (IL-1β), interleukin-6 (IL-6), interleukin-8 (IL-8), insulin-like growth factor 1 (IGF-1), VEGF, bFGF, PDGF, transforming growth factor-β (TGF-β), monocyte chemoattractant protein-1 (MCP-1), and as well as by triggering the VEGF-A signaling cascade. They are also capable of producing anti-angiogenic factors such as angiostatin and vasohibin (Bandara et al., 2017; Rezaie et al., 2019; Maacha et al., 2020). Therefore, the balance between autocrine and paracrine secretion of pro-angiogenic and anti-angiogenic factors is responsible for promoting vasculogenesis and stabilizing the newly formed vessels by adjusting the permeability of new vessels through manipulation of cell-to-cell junctions (Ghajar et al., 2006; Sorrell et al., 2009; Bussche and Van De Walle, 2014). Origin of MSCs, culture environment, culture growth factors, scaffold, and co-culture ratio can contribute to the ability of MSCs for utilization for prevascularization.

In recent years, several studies suggested the potential use of MSCs exosomes for inducing angiogenesis (Moghadasi et al., 2021). Moreover, mesenchymal exosomes extracted from the placenta and adipose tissue can be a promising tool for prevascularization (Liang et al., 2016; Komaki et al., 2017). However, there are contradictory findings regarding the angiogenic effect of BM-MSC derived exosomes. Lee et al. showed BM-MSCs’ exosomes could suppress angiogenesis (Lee et al., 2013).

MSCs can be differentiated into the endothelial lineage cells. Du et al. induced MSCs in culture with osteogenic MSCs prevascularized mesoporous bioactive glass (MBG) scaffold where they used 10 ng/ml of bFGF and 40 ng/ml of VEGF to induce endothelial differentiation in AD-MSCs. MSC-EC were positive for CD31 and vWF (Du et al., 2018). It is also reported that BM-MSCs can differentiate into ECs in a non-direct co-culture with ECs. It was observed that ECs induced differentiation of BM-MSCs into CD31 positive cells (Li et al., 2018).

### 3.2 Fibroblasts

Fibroblasts, as the main producers of ECM collagen, have an essential role in angiogenesis and wound healing process. These cells have been utilized in co-culture with endothelial cell lines such as HUVECs, EPCs, and microvascular endothelial cells (MVECs) to develop a prevascularized skin substitute (Sorrell et al., 2007; Dai et al., 2018). Fibroblasts are mostly isolated from skin, mucosal membrane and other soft tissues. Both the method and the site of isolation are considered as the important factors for the angiogenic ability of fibroblasts (Bishop et al., 1999). It was reported that formation of a capillary-like network was only observed in the culture of endothelial lineage cells with human dermal fibroblasts, not in culture with neonatal human foreskin fibroblasts 1 (hFF-1) (Costa-Almeida et al., 2014).

Since fibroblasts have mesenchymal origins, no significant marker has been identified for distinction of MSCs and fibroblasts. Markers like CD9, CD29, CD44, CD90, CD105, CD166, and CD73 are expressed in both MSCs and fibroblasts. However, there is a slight difference between the expression levels of some markers in MSCs and fibroblasts. The expression of CD106, integrin alpha 1, and IGF-2 (insulin-like growth factor 2) are high in MSCs, while MMP-1(matrix metalloproteinase-1), and MMP-3 are highly expressed by fibroblasts. Moreover, fibroblasts show a lower expression of CD146 compared with MSCs (Costa-Almeida et al., 2018).

Paracrine interactions between fibroblasts and ECs would regulate angiogenesis (Montesano et al., 1993). Physical stimulation can affect paracrine signaling, which induces angiogenesis. For instance, mechanical strain helps to stabilize capillary network assembly, and electrical stimulation can promote angiogenesis by ECs/fibroblasts interaction (Landau et al., 2018; Geng et al., 2019). In a study conducted by Asakawa et al., HUVECs cell sheet stacked with two hDFs cell sheets on a fibrin layer in five different orders. The best network formation with the highest relative vascular network lumen area was observed when HUVECs sheets directly placed on the fibrin layer and under the two fibroblast sheets (Asakawa et al., 2010). Using layer by layer assembly, Miyazaki et al. developed a prevascularized skin substitute by HUVECs and neonatal hDFs co-culture. In that study, HUVECs showed elongation at days 4 and 7 post-culture, where the vessel-like lumenized structures were clearly observed (Miyazaki et al., 2019).

HUVECs/fibroblast’s co-culture have been frequently used alongside another supporting cells or target tissue cells in different tri-culture methods. Using self-assembly and re-seeding methods, Jakubowska et al. constructed a prevascularized vaginal mucosa substitute through tri-culture of HUVECs/fibroblasts/epithelial cells, with a HUVECs/fibroblasts ratio of 2:1 (Jakubowska et al., 2020). In another study, in a tri-culture system, HUVECs and human respiratory epithelial cell were cultured with hDFs or human nasal fibroblasts in a fibrin gel and agarose-collagen type 1 scaffold which successfully formed a prevascularized respiratory mucosa.

Adding small molecules can alter network formation and morphology in an ECs/fibroblasts co-culture system. For instance, adding trehalose in a HUVECs/human normal dermal fibroblasts co-culture with a growth media containing VEGF can inhibit network formation and altering endothelial cell morphology through inhibition of VEGFR2 receptor in a dose-dependent manner. Moreover, fibroblasts in this co-culture system showed myofibroblasts’ phenotype and supported endothelial vessel network which was identified by presentation of α-smooth muscle actin (α-SMA) (Takeuchi et al., 2011). Recently, we used lacto-n-neotetraose to improve the healing process of full-thickness wounds in the mice models. Our results showed that subcutaneously injection of this oligosaccharide significantly increased the expression of VEGF in the wound bed. These findings highlight the great potential of natural materials to induce secretion of angiogenic factors within the implant sites (Farhadihosseinabadi et al., 2020).

### 3.3 Perivascular Cells

Perivascular cells including vascular smooth muscle cells (VSMCs) and pericytes regulate many features of natural vessels (Kerkar et al., 2006). VSMCs surround larger arteries, while pericytes wrap around capillaries to stabilize the newly formed vessel. VSMCs can be differentiated from mesenchymal stem cells, human embryonic stem cells, skin fibroblasts, hiPSCs or isolated from vascular tissues such as aortic ring and human umbilical cord (Montezano et al., 2017; Pang and Thomas, 2018; Perry et al., 2019). VSMCs show two different major phenotypes: synthetic and contractile. Synthetic phenotype, which is usually observed near sites of vascular remodeling, has more remarkable proliferative ability than the spindle-shaped contractile type which has less proliferative capacity and more contractile fibers (Metz et al., 2012). VSMCs isolated from microvessels and their native environment initially show spindle-shaped phenotype with a hill and valley morphology of synthetic type after growing in culture media (Kwartler et al., 2016). VSMCs in angiogenesis models are identified by α-SMA. The contractile phenotype of these smooth muscle cells is characterized by smooth muscle myosin heavy chain, smoothelin, calponin, and SM22α (Owens and Wise, 1997).

ECs and VSMCs act as a coupled system for transmission of signals from receptors localized on the endothelium surface and vice versa. Angiogenic growth factors expression will be higher when cell-to-cell interactions exist in this co-culture system (Heydarkhan-Hagvall et al., 2003). In a bilayer co-culture system, provoked by ECs-SMCs physical contacts, ECs influence SMCs’ morphology, proliferation rate, and protein production (Fillinger et al., 1993). It has been demonstrated that down regulation of PDGF expression in a spheroidal co-culture system of ECs and SMCs can inhibit apoptosis of ECs, resulting in longer viability of the vessels (Korff et al., 2001). In a study, pre-culturing of SMCs on decellularized native bone scaffold and subsequent seeding of HUVECs induced tubulogenesis. Moreover, higher vascular density and lumen formation was observed after using this method by dynamic culturing in a bio-reactor (Liu X. et al., 2018).

SMC spheroids have been used for stabilizing a mono-layer of HUVECs for developing a double-layered vascular-like structures. In that study, HUVECs were seeded around a golden needle and the encapsulated SMC spheroid were added subsequently. After 4 days of HUVECs/SMCs incubation, the bi-layer structure was separated from the needle where they showed a well-formed network vessels (Shimazu et al., 2019).

Pericytes are attached to the abluminal side of ECs, where they are around the basement membrane. These cells show excellent pro-angiogenic capabilities (Avolio et al., 2017). These cells can be harvested from umbilical cord, adipose tissue, human heart, skeletal muscle, dental pulp, saphenous vein and also from differentiation of hiPSCs (Sims, 1986; Geevarghese and Herman, 2014). Pericytes express markers such as α-SMA, NG2 (neuron-glial antigen 2), desmin, and RGS5 (regulator of G-protein signaling 5). Additionally, they represent CD73, CD90, CD105, CD146, and PDGFRβ, which are also produced by MSCs. It seems that some MSCs can be differentiated into pericytes. These differentiated cells express pericyte markers with pericyte-like morphology in ECs/MSCs co-culture, which support the endothelial network (De Souza et al., 2016a). When EPCs and MSCs were seeded on 3D polyurethane, the constructed vessels were positive for common pericytes’ markers, supporting the hypothesis that MSCs in co-culture with EPCs can be differentiated into pericytes. Furthermore, MSCs can be differentiated into pericytes after subsequent addition into a pre-formed capillary-like network (McFadden et al., 2013). Similarly, pericytes have the ability to be differentiated to the other mesenchymal-originated cells such as SMCs, fibroblasts and osteoblasts that can significantly alter metabolic and mechanical properties of these supporting cells as well as signaling cascades of ECs (Armulik et al., 2005; Bergers and Song, 2005; Blocki et al., 2013; Orlova et al., 2014).

While ECs/pericytes in a 3D co-culture can result in tube-like formations, a 2D co-culture with ECs/pericytes direct contact does not form such structures (Darland and D’amore, 2001). Several studies indicated that pericytes might act as inhibitors of ECs proliferation. It has been suggested that pericytes prevent proliferation of ECs via contact inhibition. Although, they inhibit proliferation of ECs, they stabilize the forming vessels and alter tube length lumen diameter and sprouting (Orlidge and D’amore, 1987; Waters et al., 2013).

Pericytes have been used in tri-culture system in the presence of HUVECs and hiPSC-MSCs with a HUVECs/pericytes ratio of 4:1 for prevascularizing a calcium-phosphate scaffold. In that investigation, both angiogenesis and osteogenesis were significantly promoted after implantation the developed construct in the rat cranial bone defect (Zhang C. et al., 2017).

Pericyte-like MSCs and SMCs can support the newly formed endothelial cell network in culture with EPCs (Loibl et al., 2014). Cell sheets engineered by EPCs and SMCs differentiated from MSCs improved ejection fraction of infarcted cardiac muscles (Shudo et al., 2017). It is reported that prevascularization of a polystyrene scaffold was achieved by co-culture of EPCs and SMCs with a 6:1 ratio (Jia et al., 2018a).

### 3.4 Differentiated Cells in Target Tissues

The native cells of the target tissue can indirectly affect differentiation and proliferation of ECs and development of the capillary-like networks. Besides, vascular development can affect the tissue-resident cells behaviors. These reciprocal interactions can influence the formation of prevascularized target tissues such as neural networks. HUVECs in co-culture with Witsar rat neural cells by a ratio of 1:2.5 were used for prevascularization of a poly (L-lactide) (PLLA)/poly (lactide-co-glycolide) (PLGA) scaffold. It was observed that interaction between endothelial cell network and neural cells lead to development of a more complex neural network and morphologies (Shor et al., 2018). Vascular growth can interfere with development of myotubules formations. HUVECs in culture with skeletal muscle cells isolated from fresh human muscle tissue were used for prevascularization of skeletal muscle constructs. In that study, two methods have been evaluated. In the first method, direct co-culture of HUVECs and skeletal muscle cells were considered for developing a prevascularized construct, while in the second method myotubules differentiated in a HUVECs free environment and endothelial cells in a fibril hydrogel were subsequently added. The findings indicated that in the first method, myotubules development was suboptimal and endothelial network development interfered with myotubules differentiation. However, subsequent addition of endothelial cells resulted in better capillary network development without interfering with myotubule differentiation (Gholobova et al., 2020).

The development of vascular network does not always hinder differentiation of target tissue cells and even sometimes endothelial cell differentiation can enhance capabilities of target tissue cells and promote target tissue cell proliferation. Using layer by layer assembly, HUVECs in culture with human hepatocytes and fibroblasts were used for developing a 3D liver engineered tissue construct. After implantation, prevascularized construct produced more albumin, which indicates that prevascularization can enhance performance of hepatocytes (Sasaki et al., 2017). In another study, a sub-population of EPCs, outgrowth endothelial cells (OECs) from peripheral blood, cultured with human primary osteoblasts were used for prevascularization of platelet-rich fibrin (PRF). It was reported that capillary-like structures were developed 7 days post-culture and primary osteoblasts promoted angiogenic abilities of EPCs (Dohle et al., 2018).

Several strategies such as adding small molecules, culturing under hypoxic condition and subsequent seeding of endothelial cells have been considered to promote ECs/target tissue cells interactions. Co-culture of HUVECs and human endometrial epithelial cells with a 1:1 ratio in a 3D collagen scaffold was successfully used for developing an endothelial network. It was showed that adding estradiol could not influence endothelial cell behaviors directly, however, estradiol increased endogenous VEGF levels of endometrial epithelial cells which promoted endothelial network formation (Pence et al., 2015).

Short term hypoxia improves development of a capillary-like network in an ECs/primary osteoblasts co-culture. However, prolonged hypoxia is considered as a cytotoxic parameter that shows negative effect on capillary network formation. (Gholobova et al., 2020) Sometimes, ECs can be used with heterogeneous populations of stem cells for fabricating a capillary-like network. HUVECs in co-culture with amniotic fluid stem cells were utilized for prevascularization of a collagen chondroitin sulfate scaffold. Culturing under hypoxic conditions did not result in better capillary formation development. It was revealed that hypoxia increased VEGF, PDGF, and VEGR1, but reduced expression of VEGFR2 (Lloyd-Griffith et al., 2015). In some studies, instead of using an isolated cell line, cellular extract or isolated heterogeneous cell population or isolated microvessels were used for prevascularizing scaffolds. For instance, stromal vascular fraction (SVF) (isolated from adipose tissue which contains mesenchymal cells, endothelial progenitor cells, hematopoietic lineage cells and stromal cells, fibroblast and pericytes) and micro vascular fraction (MVF-microvascular fragments that are usually isolated from adipose tissue) can be used for prevascularization of engineered tissue constructs. It has been shown that wound healing can be promoted by encapsulating SVF cells in a collagen-fibrin hydrogel (Nilforoushzadeh et al., 2019). In another study, a prevascularized adipose tissue was constructed by using MVF (Acosta et al., 2020). Furthermore, a prevascularized skin substitute was developed utilizing MVF and SVF via *in vivo* prevascularization methods (Später et al., 2018). In another study amniotic fluid stem cells cultured with HUVECs with a ratio of 4:1 resulted in prevascularization of a collagen chondroitin sulphate scaffold in 7 days (Lloyd-Griffith et al., 2015).

## 4 Co-culture System Optimization

Providing a proper environment for proliferation and migration of endothelial cells, and development of a capillary-like network assembly with the help of supporting cells can be somewhat challenging since many considerations including the following parameters must be taken into account (Supplementary Table S1).

### 4.1 Target Tissue

**FIGURE 2:**
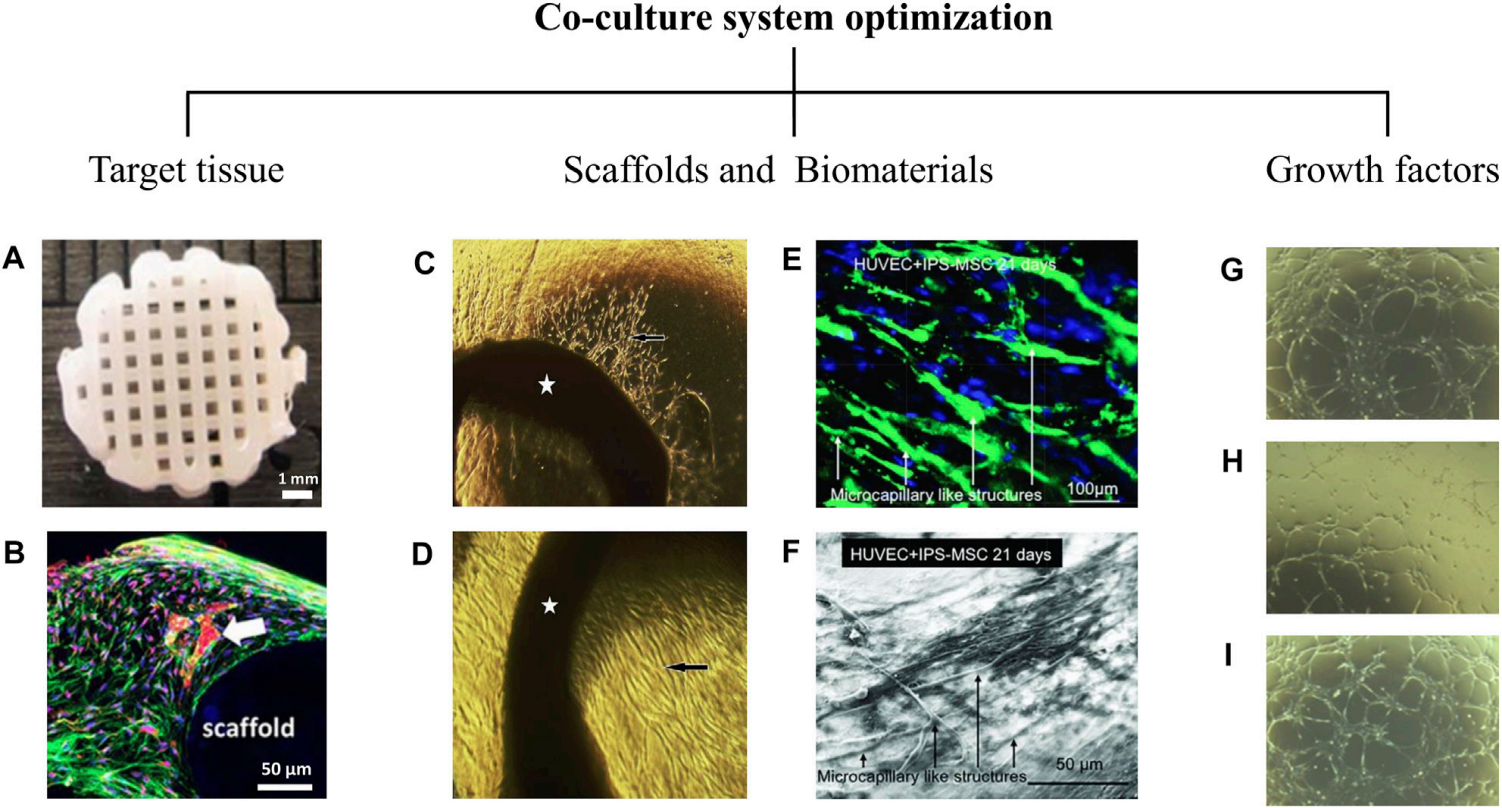
Co-culture system optimization. Co-culture system optimization depends on critical factors that profoundly alter cell behaviors. Target tissue is a decisive factor in the vascularization process. The cellular microenvironment is another critical factor that influences prevascularization. As shown in the **(A,B)**, co-culture of AD-MSCs (Adipose tissue derived mesenchymal stem cells) and HUVECs (Human umbilical vein endothelial cells) in a 3D printed polycaprolactone/hydroxyapatite scaffold coated with cell-laden hydrogels leads to the formation of an appropriate capillary network (white arrow) at day 21 (Kuss et al., 2018). It is evident that topographical and physicochemical properties and even the type of biomaterial used in scaffolds affect prevascularization. **(C,D)** Aortic rings (pointed by stars) cultured on the mesenchymal side **(C)** and epithelial side **(D)** of a decellularized amniotic membrane developed in our laboratory. After 7 days of culture, capillary-like structures and endothelial-like penetrations (black arrows) have been identified on both sides of the amniotic membrane (original magnification X40) (Niknejad et al., 2013). **(E,F)** HUVECs and hiPSC-MSC (human induced pluripotent stem cell derived mesenchymal stem cells) co-culture on a calcium phosphate cement (CPC) scaffold. Microcapillary formations was formed at day 21 (Liu et al., 2017). The prevascularization process is also influenced by growth factors used in cell culture media. **(G–I)** The formation of cord-like structures by endothelial cells in response to FGF2 **(G)**, VEGF **(H)**, and the combination of both **(I)** has shown at day 10 (original magnification X20) (Khan et al., 2017).

Since blood perfusion and vascular network of most of the tissues are different, it is evident that target tissue can play a significant role in determining the best types of endothelial and supporting cells for the co-culture system as well as methods and the other co-culture factors (Figures 2A,B). The structure of target tissues is a decisive factor in choosing the method of prevascularization. For instance, 2D co-culture systems and cell sheet engineering can be used for developing the prevascularized 2D constructs such as cardiac patch, skin graft, or lumenized structures including vascular or urethral grafts. In contrast, cell spheroids and cell encapsulations can be used for developing a 3D bone constructs. Moreover, since formation of a vascular network can change the biomechanical properties of scaffolds, many studies have been conducted to develop prevascularized scaffolds without reducing their biomechanical properties. Considering the biomechanical properties of target tissues, an appropriate scaffold can be selected to limit the uncontrolled formation of vascular-like structures.

The formation of a vascular network can impact some unique features of target tissues. For example, it has been shown that although the optimal vascular network development was seen in HUVECs/MSCs co-culture systems, the mineralization of the scaffolds was decreased in the co-culture group compared with MSCs monoculture (Ma et al., 2014b). In another example, increasing the cell seeding density of fibroblasts enhanced the mechanical properties of a prevascularized cardiac patch, while it decreased cardiac conductivity. Furthermore, the target tissue cells can significantly impact the formation of vessels by either promoting or inhibiting vasculogenesis and interacting with endothelial cells (Ong et al., 2017).

### 4.2 Scaffolds and Biomaterials

The crosstalk between ECM components and ECM composition such as collagen type I and IV, laminin, fibrin, and hyaluronic acid have a direct effect on ECs migration and elongation, length and number of sprouts as well as the alignment of newly formed vessels in the natural process of angiogenesis and vasculogenesis. The scaffolds mimic ECM by providing an environment that can support endothelial attachment, sprout formation, lumenogenesis and tubulogenesis and finally regulate vasculogenesis process (Figures 2C–F). Although, the currently designed 3D scaffolds are less suitable to be applied *in vivo* and the ideal strength is not achieved yet, there are many improvements in cell penetration and migration inside the tissues thanks to bioprinting technology. Factors influencing scaffold design vary from case to case. To design a prevascularized implantable and viable tissue, the scaffold should be permeable and able to excrete wastes out of the tissue. Moreover, scaffolds are needed to show appropriate biocompatibility and less toxicity. Natural biomaterials are derived from components found in extracellular matrix, such as collagen or other natural materials including those obtained from plants, insects, or animals. Natural biomaterials usually have superb biocompatibility with the binding sites for proteins and cells similar to ECM. We used a Gelatin Methacryloyl (GelMA)/chitosan nanoparticles composite hydrogel to delivery bFGF angiogenic factor. According to our findings, the bFGF was released from the developed scaffold in a sustain manner and promoted proliferation of cultured fibroblasts. The GelMA/chitosan nanoparticles scaffold exhibited an excellent biocompatibility with great potential to be used in prevascularization processes (Modaresifar et al., 2018). However, natural materials may be immunogen and suffer from limited physical and mechanical stability. Major advantages of synthetic biomaterials include control over the bio-chemical and physical characteristics, and degradation rate of these constructs. Besides, these polymers are highly tunable and their pore size can be controlled for enhancing angiogenesis. To come up with a scaffold that take advantage of both biomechanical properties of a synthetic scaffold and cell supporting abilities of natural scaffolds, synthetic biomaterials are frequently coated with natural ones (Yu et al., 2015).

### 4.3 Growth Factors and Culture Conditions

Adding angiogenic factors to tissue-engineered constructs can enhance their vascularization process (Figures 2G–I) (Patel et al., 2008). Pro-angiogenic growth factors are used to initiate different steps of angiogenesis. Formation of new vessels can be increased by direct or indirect role of growth factors. VEGF and bFGF show direct effect on angiogenesis (Choi et al., 2018; Kawecki et al., 2021). They stimulate mobilization of EPCs that speed up the initiation of angiogenesis. For instance, it has been reported that genipin cross-linked electrospun gelatin mats loaded with VEGF can stimulate and induce angiogenesis for tissue engineering applications (Del Gaudio et al., 2013). However, the concentration of angiogenic factors must be firmly controlled because severe vascular leakage or hemangioma may occur in high concentrations of VEGF.

In addition to main angiogenic factors, there are some growth factors that indirectly increase angiogenesis process. These products including sonic hedgehog homolog (SHH) (Weyers et al., 2020), HIF-1 (Chai et al., 2020; Coyle et al., 2020), and BMP (Samee et al., 2008; Yang et al., 2021), which recruit cells in the vascularization area to secret angiogenic factors. PDGF, angiopoietin 1, and ephrinB2 also show an indirect role on angiogenesis (Harel et al., 2020; Hosaka et al., 2020; Yoon et al., 2020). They may help to stabilize the newly formed capillaries by recruiting smooth muscle cells. VEGF initiate angiogenesis but cannot induce blood vessel maturation, while PDGF induce blood vessel stability and maturation. Furthermore, combined administration of growth factors such as VEGF/bFGF (He et al., 2012) and VEGF/PDGF (Lutton et al., 2012) have shown an effective way to generate more stable capillary bed (Wang B. et al., 2020). However, further studies on the combined use of angiogenic agents help to identify the optimal angiogenic environment used in various fields of tissue engineering.

Hypoxic conditions can enhance the angiogenic ability of MSCs in the culture media. The MSCs cultured under hypoxic condition showed higher expression of pro-angiogenic factors including VEGF, vWF, FGF, and Flk-1, which significantly improved vascularization of PLGA scaffold compared with MSCs cultured under normal conditions (Kim et al., 2019). Confirmed by another study, MSCs sheet engineered under hypoxic conditions (2% O_2_) had greater capillary network development compared to those cultured under normal condition (20% O_2_) (Zhang et al., 2016).

### 4.4 Prevascularization Strategy

One of the critical elements determining the quality of prevascularization is choosing the most suitable method. The most important consideration for selecting the most suitable method is the target tissue. 2D prevascularization methods can be used for prevascularizing tissues like skin, gastrointestinal and urethral tract. Direct cell seeding on a 2D scaffold is one of the most common, yet inefficient methods for the prevascularization of 2D engineered constructs since random incorporation of endothelial lineage cells and supporting cells does not result in an aligned, maintainable, and functional vascular-like network. Co-culture of cells on highly aligned microfibers or using bioprinting for the patterned culture of endothelial cells and supporting cells on the scaffold have been proposed as potential solutions for this issue (Bourget et al., 2016; Qian et al., 2019).

Cell culture in two dimensions has been routinely performed during past decades in many labs. Although this approach was considered suitable, it does not properly mimic that of natural tissues function (Jensen and Teng, 2020). There are different cell responses in two and three-dimensional cultures. Prevascularization of 3D tissues can be much more challenging since it requires the endothelial network assembly to be aligned in a three-dimensional space. Some cells will immediately lose their normal physiologic features after they are taken out of the body and placed in 2D cell culture, while they might not be affected in a 3D culture medium (Duval et al., 2017). Both *in vivo* and *in vitro* strategies have been employed to achieve a functional 3D endothelial network assembly.

*In-Vivo* prevascularization methods such as AV-loop use the angiogenesis ability of host vessels to penetrate and vascularize the scaffold. In the AV-loop method, an arteriovenous shunt is surgically incorporated into the scaffold in order to prevascularize the construct (Wu et al., 2017; Weigand et al., 2018). Another *in vivo* strategy is implantation of the scaffold in a highly vascular pouch or flap in the body of the host (Lee et al., 2021). After the prevascularization of the scaffold, the final scaffold can be implanted in the defected tissue. However, these methods are time-consuming and invasive and are not ideal for translation in clinical practice.

Over the years, direct injection of endothelial cell suspensions has been the most frequent strategy applied in regenerative medicine. This simple method has many weaknesses, including rapid diffusion of cells, low engraftment efficiency by loss of ECM interactions, and shear-induced cell death (Kito et al., 2013). Although random seeding of endothelial cells with supporting cells in a 3D scaffold with an optimal ratio in a biomaterial with optimal pore size can result in an endothelial network assembly, this method requires a lot of trial and error, and usually, the fabricated network is not organized. Furthermore, another issue is survival, differentiation, and migration of incorporated cells in a 3D environment. Encapsulating endothelial lineage cells and supporting cells in hydrogels containing essential factors for survival and differentiation of cells such as VEGF can ensure the survival of incorporated cells (Phelps et al., 2015; Kuss et al., 2018). Furthermore, it has been shown that using bioreactors for a dynamic culture environment with chemical and mechanical stimuli or growth factors gradient can direct the alignment of endothelial network assembly formed by encapsulated endothelial lineage cells/supporting cells (Tocchio et al., 2015; Nguyen et al., 2017).

Using cell spheroids as a prevasculrizing unit is an option to overcome the limitations of routine cell suspension methods. The endothelial cell spheroid system was first established as an *in vitro* model to study endothelial cell differentiation (Korff and Augustin, 1998). The spheroid sprouting method has also been applied as one of the *in vitro* models of angiogenesis. However, investigations on its ability to create a prevascularized tissue are in progress (Blacher et al., 2014). One study investigated bone regeneration using the co-culture of endothelial cells and osteoblasts for vascular sprouting and angiogenesis initiation. It was demonstrated that this co-culture system is likely to control the angiogenic properties of the ECs and also has a role in the optimization of osteoblast differentiation (Stahl et al., 2004). Encapsulation of cell spheroids has also been used for prevascularization. It has been shown that encapsulation of cell spheroid can result in more intricate and organized vascular network development compared to routine cell encapsulation (De Moor et al., 2021; Roux et al., 2021). However, it is worth noting that optimal EC/supporting cell ratio and EC/supporting cell interactions can be quite different in the spheroidal environment compared to the 2D and 3D co-culture systems (Benmeridja et al., 2020).

Scaffold-free cell sheet engineering has been widely used to achieve prevascularized 2D and 3D tissue constructs. Cell sheet engineering is a method of developing tissue constructs in which cell sheets are usually harvested on temperature-sensitive culture dishes (Yamato and Okano, 2004). These dishes are made by covalent grafting of a temperature-responsive polymer to ordinary culture plates. Changing the temperature modifies the surface characteristics from hydrophilic to hydrophobic allowing many cells to attach or detach. Alternatively, instead of thermal fluctuation, electromagnetic force can be utilized for attaching and detaching magnetic responsive sheets (Silva et al., 2020). In both methods, the formed cell sheets can keep their ECM and thus freely stick to other surfaces, including host tissues and another cell sheet. Several, tissues and organs such as the skin, the cornea, urothelium, and cardiac muscle are reconstructed by this method (Ng and Hutmacher, 2006; Umemoto et al., 2013; Song et al., 2020). One of the drawbacks of cell sheet engineering is ischemia or hypoxia of cells in the core. It has also been demonstrated that the location of the endothelial cell sheet compared to sheets composed of supporting cells could be crucial for developing an organized endothelial network and preventing hypoxia (Asakawa et al., 2010).

Recent advances in 3D-bioprinting, micro-molding, and microfluidic enabled researchers to micro-design the scaffold. As mentioned, micro-designing of the scaffold and micro-patterning of the surface topography has been used as one of the leading solutions to achieve an aligned network. Currently, lab-on-a-chip technologies have been widely used as angiogenesis and vasculogenesis models *in vitro* (Ko et al., 2019). These strategies focus on fabricating microchannels that will be later cellularized by endothelial and supporting cells to achieve a prevascularized construct. Several techniques such as microfluidics, laser degradation, photolithography, layer by layer assembly, micro-molding, and biodegradable sacrificial-template have been used to make microchannels mimicking the natural vascular network.

Microfluidic techniques have been widely used for simulating vasculogenesis and angiogenesis (Rambøl et al., 2020). One of the main challenges regarding the use of the microfluidic technique is finding the correct factors to maintain the optimal flow for cellularizing channels with endothelial and supporting cells. Furthermore, the microfluidic techniques are not scalable, and currently, most of their application remains limited to angiogenesis models. A common method for micropatterning is lithography which uses a particular electromagnetic wavelength to fabricate specific patterns on a scaffold incorporated with photosensitive biomaterials (Michna et al., 2018). An aligned microchannels network can be fabricated using this technique that will be used for the adhesion of endothelial cells to form an intricate and aligned endothelial network (Jiang and Luo, 2013). Another method of developing a microchannel network is using a biodegradable sacrificial template that can be degraded or washed after implantation and forming the microchannel network (Miller et al., 2012; Li et al., 2017; Hu et al., 2021). The microchannels can also be formed using laser biodegradation techniques (Pradhan et al., 2017). Another method is the layer-by-layer assembly technique which is used to form a thin scaffold with various patterns. In this technique, alternative charged layers will be deposited layer-by-layer to form microchannels (Sousa et al., 2021). These microchannels can be used as substrates for adhesion, proliferation, and network formation of endothelial cells (Miyazaki et al., 2019). These methods provide great control over the alignment of vascular-like networks. However, currently, the choice of biomaterials for designing the scaffold is limited, which means that the biomaterials used for creating these microchannels do not provide the ideal niche for endothelial cells. Also, we have yet to achieve 3D constructs with microchannels with desirable biomechanical properties using these techniques. Furthermore, inosculation of microchannels formed by these techniques to the host vessels after implantation requires further *in vivo* investigations. Overall, these advances are promising, and further investigations in this area can pave the way for achieving a 3D engineered tissue with an organized and functional vascular network.

Another solution for fabricating a 3D prevascularized engineered construct is 3D bioprinting of endothelial and supporting cells or EC/supporting cell spheroid in a similar pattern to the vascular network (De Moor et al., 2021). This method can be used in combination with other techniques to achieve proper results (Nulty et al., 2021). However, one of the main obstacles of bioprinting is ensuring the survival of cells through the process of bio-printing. Benning et al. investigated the ability of various hydrogels including Matrigel, collagen, gelatin, gelatin/alginate, fibrin, agarose, and Pluronic F-127 for bioprinting of endothelial cells. They observed that endothelial cells that have been bioprinted using collagen and fibrin hydrogels were capable of proliferation and sprouting and maintained their endothelial phenotype. Although endothelial cells bioprinting in gelatin hydrogels were capable of proliferation, they were unable to initiate sprouting (Benning et al., 2018). Due to the promising results of bioprinting, developing more suitable hydrogels can be essential for using bioprinting in prevascularization.

## 5 Future Perspective

*In vitro* prevascularization strategies emphasize on optimization of the interactions between cells, biomaterials and culture conditions. To date, a wide variety of cells with diverse vasculogenic features have been studied to ensure survival of the engineered tissue. HUVECs, taking advantages of convenient and ethical isolation methods, are the most common endothelial cell lineage employed in prevascularization strategies and angiogenesis methods. Furthermore, efforts are being made to find a suitable cell source with high accessibility and angiogenesis capability. In this light, MSC-ECs and hiPSC-ECs have been suggested for the prevascularization of scaffolds since they can easily be isolated from various tissue, differentiated *in vitro*, and act as an autologous cell source. However, several inconsistencies exist in the reports regarding their pro-angiogenic abilities and their utilization for prevascularization. Further studies must be conducted to compare the pro-angiogenic capabilities of these cells, their markers, and their morphology to the current common endothelial lineage cells used in prevascularization. Moreover, amniotic-derived cells seem provide a reliable cell source that can be easily isolated and differentiated toward endothelial cells (Yazdanpanah et al., 2015; Abbasi-Kangevari et al., 2019). These cells also produce various angiogenic growth factors that candidate them as an appropriate supporting cell. Beside the cell sources, advances in biomaterial designs come up with positive results in prevascularization. Recent studies have focused on new strategies such as cell bioprinting and producing highly-tunable scaffolds with micro-patterning design to make further organized vessels similar to host vascular hierarchy. However, an important roadblock in achieving a sustainable vascularized construct is the interference of endothelial network development with the target tissue cells. To overcome this challenge, different ways are available including changing the ratio of endothelial cells and protective cells, applying genetic modifications to alter the signaling pathways in favor of angiogenesis, and using different growth factors and small molecule to facilitate anastomosis in the target tissue. Future studies provide more tools for achieving perfect prevascularization approaches to develop functional tissue engineered constructs.
