# Supplementary material for: Recent Advances on Cell-Based Co-Culture Strategies for Prevascularization in Tissue Engineering
Source: Front Bioeng Biotechnol. 2021 Nov 25;9:745314. doi: 10.3389/fbioe.2021.745314 (PMC8655789; doi:10.3389/fbioe.2021.745314)
Supplement: Supplementary file 1 [file Table1.docx]

**Supplementary Table 1:** A summary of studies on endothelial/supporting cells co-culture systems.

Cells; HUVEC: Human umbilical vein endothelial cell; MSC: Mesenchymal stem cells; AD-MSC: adipose tissue derived Mesenchymal stem cells; BM-MSCs: Bone marrow derieved mesenchymal stem cell; hESC: human embryonic stem cells; hiPSC: Human induced pluripotent stem cell; EC: endothelial cells; SMC: smooth muscle cells; EPC: endothelial progenitor cell; FTM HUCPVC: First trimester human umbilical cord perivascular cells; hPAEC: Human pulmonary artery endothelial cells; hCECs: human cardiac endothelial cells; hCMVEC: human Caardiac microvasclar endothelial cell; HDMEC: human dermal microvascular endothelial cell; AFSCs: amniotic fluid stem cells; NHDF: Neonatal human dermal fibroblasts; Materials; CPC: calcium phosphate cement; PLLA: poly(l-lactic acid); PLGA: Poly (lactic-co-glycolic acid); PCL: polycaprolactone; PDLA: poly(d-lactic acid); PES/PVP: polyethersulfone/polyvinylpyrrolidone; TEDG: tissue engineered dermal graft

| **Endothelial lineage cells** | **Supporting cells** | **Culture method** | **Biomaterials and scaffolds** | **Seeding ratio** | **Target and In-Vivo experiment** | **ref** |
| --- | --- | --- | --- | --- | --- | --- |
| HUVECs commercial cell line | Adipose tissue-derived MSCs | Cell seeding | Porous titanium fiber mesh | HUVECs 1:1 MSCs | Cranial defect in rat | (Ma et al., 2014) |
| Human umbilical vein HUVECs | Bone marrow  Umbilical blood  hESC (human embryonic stem cell)  hiPSC MSCs | Cell seeding | - | Macroporous and biofunctionalized CPC | Bone | (Chen et al., 2018) |
| HUVECs Commercial cell line | hiPSCs-derived MSCs | Cell seeding | Calcium phosphate cement | - | Cranial defect in rat | (Liu et al., 2017) |
| Human umbilical vein HUVECs | Human adipose derived- MSCs | Layer by layer cell deposition  by laser assisted bioprinting | HUVECs 1:1 MSCs | Osseous sheets | Bone | (Kawecki et al., 2018) |
| HUVECs commercial cell line | Bone marrow-derived MSCs | Compared Static and dynamic culture condition | - | Collagen hydrogel  alginate | - | (Nguyen et al., 2017) |
| Human umbilical vein HUVECs | Fibroblasts obtained from Human nasal dermis /  MSCs obtained from Wharton jelly,  adipose tissue, and  bone marrow | Cells were entrapped into the hydrogel during the molding process | - | Fibrin, Agarose-Collagen | - | (Kniebs et al., 2020) |
| Human umbilical vein HUVECs/  Human umbilical cord blood EPCs | Human Aorta derived-SMCs | Cell seeding | ECs 1:4 SMCs | Cell adhesive polyethylene glycol | General | (Peters et al., 2016) |
| human bone marrow-derived EPCs | MSCs (FTM HUCPVCs) | Cell seeding | EPCs 1:2 MSCs and  EPCs 1:4 MSCs | Matrigel | - | (Iqbal et al., 2017) |
| Human umbilical cord blood EPCs | Myocardial cell | Cell seeding | - | - | Cardia | (Rosca et al., 2018) |
| Human peripheral blood EPCs/  Human umbilical vein HUVECs | Osteoblast cell line MG63 | Subsequent seeding of endothelial cells | ECs 2:3 Osteoblasts | - | Bone | (Fuchs et al., 2007) |
| Human umbilical cord blood EPCs | Human foreskin dermis-derived fibroblasts  and keratinocytes | Cell seeding | Human plasma/calcium chloride gel |  | Nude mice skin wound model | (Dai et al., 2018) |
| EPCs  obtained from Bone marrow and peripheral blood of Dutch milk goat /  Goat vein endothelial cells obtained from Jugular vein of Dutch milk goat | MSCs obtained from Iliac bone of goat | 2D co-culture in Matrigel  / 3D co-culture by encapsulation in Matrigel | EPCs 1:1 MSCs | Matrigel tm  Fibronectin | Bone | (Fedorovich et al., 2010) |
| Peripheral blood EPCs | Oral mucosa fibroblasts and keratinocytes | Cell sheet | - | - | Excisional wound in nude mice | (Lee et al., 2019) |
| HUVECs  hPAECs hCECs  ECs | Human stromal cells obtained from human adipose tissue | Cell seeding | - | Matrix of decellularized porcine small intestinal submucosa (SIS)  Matrigel, collagen | - | (Manikowski et al., 2018) |
| Differentiated endothelial cells obtained from Bone marrow MSCs of Witsar rats | MSCs from Bone marrow of Witsar rat | Cell sheet | - | Fibroblast nichecoated  TEDG | Critical-sized calvarial bone defect in rat | (Xu et al., 2019) |
| Human cardiac microvascular hCMVECs | hMSCs/Embryonic cardiomyocytes differentiated from hiPSC | Cell seeding | - | Collagen cell carrier | Cardia | (Valarmathi et al., 2017) |
| HDMECs from human foreskin dermis | Gingiva fibroblasts/  Gingiva epithelial cells | Cell seeding | HDMECs 1:1 Fibroblasts | - | Urethra subcutaneous implantation | (Heller et al., 2016) |
| Endothelial cells obtained from pluripotent cells of rat embryo | Derm fibroblasts/cardiomyocytes from Embryonic induction | 3D suspension culture  And cell sheet | - | - | Cardia | (Masuda et al., 2015) |
| Commercial cell line HUVECs  and  HDMVECs | Human dermis Fibroblast  And Bone marrow-derived MSCs | Cell seeding | - | - | Skin | (Sorrell et al., 2007) |
| Rat bone marrow derived-EPCs | Rat bone marrow derived-MSCs | Cell seeding | EPCs 1:1 MSCs | Matrigel | - | (Aguirre et al., 2010) |
| HUVECs commercial cell line | Proximal tubular epithelial cells (RPTEC)  /GEC from kidney of rat | Cell seeding | - | - | Kidney | (Kim et al., 2002) |
| HUVECs commercial cell line | Human Cancellous bone osteoblasts | - | HUVECs 1:1 Osteoblasts | Biodegradable polyurethane scaffold | Bone | (Hofmann et al., 2008) |
| HUVECs commercial cell line | Human dermis fibroblasts | 3-layer cell sheet | HUVECs 1:2 Fibroblasts |  | Skin | (Asakawa et al., 2010) |
| HUVECs commercial cell line | Human Aorta derived-SMCs | Bioreactor, cell seeding | Subsequent seeding of HUVECs after 3 weeks | CPC  Decellularized porcine scaffold | Bone | (Liu et al., 2018b) |
| HUVECs commercial cell line | Human aorta SMCs | Encapsulation of cell spheroids | - | Photo-cross-linkable gelatin methacrylate | Large vessels | (Shimazu et al., 2019) |
| Human umbilical vein HUVECs | MSCs  Pericytes differentiated from hiPSCs  Commercial cell line | Cell seeding | HUVECs 4:1 pericytes | Calcium phosphate cement | Cranial bone defect in nude rat | (Zhang et al., 2017) |
| Rat bone marrow-derived EPCs | Rat bone marrow- derived MSCs differentiated toward SMCs | Cell seeding | EPCs seeded with a density of 1.5×10^5^/cm^2^ on a confluent SMC culture on fibronectin | - | Ischemic-induced heart defect in rat | (Shudo et al., 2017) |
| EPCs obtained from New Zealand rabbit Peripheral blood | SMCs obtained from bladder of rabbit | Cell sheet | EPCs 1:6 SMCs | - | Implanted subcutaneously on Dorsal skin of rat | (Jia et al., 2018) |
| HUVECs commercial cell line | Witsar rat  neural cells | Cell seeding | HUVECs 1:1 Neural cells | PLLA/PLGA | Spinal chamber Neurovascular | (Shor et al., 2018) |
| Human umbilical vein  HUVECs | Endometria Epithelial cells | Cell seeding | Epithelial cells 1:01 endothelial | Cross-linked collagen scaffold | Uterus | (Pence et al., 2015) |
| Human umbilical vein HUVECs | AFSCs (Amniotic fluid stem cells) | Cell seeding | ECs 1:4 AFSCs | Collagen chondroitin sulphate | Bone and thick tissues | (Lloyd-Griffith et al., 2015) |
| Human umbilical vein HUVECs | Human dermis fibroblasts | Cell seeding | HUVECs 5:1 Fibroblasts | Porous silk fibroin films | Skin | (Zhu et al., 2018) |
| HUVECs commercial cell line | Fibroblast and Keratinocytes commercial cell lines | layer by layer assembly | NHDFs and HUVECs seeding ratios were 1,000:1, 500:1, or 100:1, and subsequently keratinocytes seeded with a 1:100 ratio |  | Excisional wound splinting model in mice | (Miyazaki et al., 2019) |
| HUVECs commercial cell line | MSCs Commercial cell line | Cell spheroid  encapsulation | HUVECs 1:4 MSCs | Collagen fibrin | Bone | (Heo et al., 2019) |
| HUVECs commercial cell line | Human apical papilla-derived MSCs | Laser assisted bioprinting | Rat tail collagen type 1 | - | Calvarial bone defect in the rat model | (Kérourédan et al., 2019) |
| Rat bone marrow derived-EPCs | Rat bone marrow derived-MSCs | Subsequent seeding of EPCs on MSCs cell sheets | - | - | Tibial defect in rat | (Liu et al., 2018a) |
| HUVECs commercial cell line | Rat bone marrow- derived MSCs | Subsequent seeding of MSCs porous silk fibroin scaffold. HUVECs were previously seeded on scaffold | - | Porous silk fibroin | Dorsal subcutaneous pockets in mice | (Zhang et al., 2015) |
| HUVECs commercial cell line | Human bone marrow- derived MSCs | Uses Two different aggregation techniques before encapsulation | HUVECs 5:95 MSCs | Rat tai collagen type 1 | Bone | (Heo et al., 2019)  (Deegan et al., 2019) |
| HUVECs commercial cell line | Human bone marrow MSCs | Cell seeding | - | Polycaprolactone (PCL) micro-fiber constructs | Calvarial bone defect in rat | (Freeman et al., 2020) |
| Human umbilical vein HUVECs | Human bone marrow-derived MSCs | Cell seeding | - | PCL-alginate | Bone | (Freeman et al., 2015) |
| HUVECs commercial cell line | - | Bioprinting | - | PLGA/Hydrogel Biopapers | - | (Pirlo et al., 2012) |
| hMSCs-derived endothelial cells | Adipose-derived MSCs/  MSC-derived Fibroblasts | Cell seeding | - | Fibroblast niche coated  TEDG | Skin | (Ajit et al., 2020) |
| HUVECs commercial cell line | Human dermis fibroblasts | Cell seeding on scaffold with an organized surface tomographical features | - | PES/PVP | Soft tissues | (Skrzypek et al., 2018) |
| Vascular endothelial cell (VECs) obtained from descending aorta of rat | Rat bone marrow-derived BMSCs | Noncontact co-culture | BMSCs 1:1 VECs | - | Bone | (Jiang et al., 2018) |
| HMCEC human Immortalized microvascular cells | Human -derived one marrow MSCs | Cell seeding | - | PLLA/PDLA sheet | Bone | (Cipriano et al., 2020) |
| HUVECs commercial cell line | Human vagina fibroblasts/  Human vagina Epithelial cells | Self-assembly  reseeding | HUVECs 1:2 Fibroblasts | - | Subcutaneous implantation on back of a nude mice | (Jakubowska et al., 2020) |
| HUVECs commercial cell line | Rabbit bone marrow-derived MSCs | Cell sheet | - | - | Nerve conduit | (Fan et al., 2020) |
| HUVECs commercial cell line | MSCs Commercial cell line | Cell sheet | HUVECs 2:1 MSCs | - | Rat full thickness skin wound model | (Chen et al., 2017) |
| Human umbilical vein HUVECs | SMCs from human muscle biopsy | Cell seeding  and delayed seeding of Endothelial Cells | - | - | Implanted on Fascia of Latissimus Dorsi | (Gholobova et al., 2020) |
| Human umbilical vein HUVECs / hiPSCs-derived  Endothelial cells | Adipose tissue derived- MSCs/ Lung fibroblasts | - | ECs 5:1 MSCs/Fibroblasts | Pancreatic island of rat | Pancreas | (Rambøl et al., 2020) |
| HUVECs commercial cell line | Human adult heart ventricule fibroblasts/Cardiomyocytes differentiated from hiPSCs | 3D bioprinting of cell spheroids | (CM:FB:EC 70:15:15, 70:0:30, 45:40:15) | - | Cardia | (Ong et al., 2017) |

# References

Aguirre, A., Planell, J., Engel, E.J.B., and communications, b.r. (2010). Dynamics of bone marrow-derived endothelial progenitor cell/mesenchymal stem cell interaction in co-culture and its implications in angiogenesis. 400(2)**,** 284-291.

Ajit, A., Ramakrishnan, R., Retnabai, S.T., Senan, M., and Krishnan, L.K.J.J.o.B.M.R.P.B.A.B. (2020). Generation of niche tuned antifibrotic fibroblasts and non‐viral mediated endothelial commitment using adipose stem cells for dermal graft development. 108(7)**,** 2807-2819.

Asakawa, N., Shimizu, T., Tsuda, Y., Sekiya, S., Sasagawa, T., Yamato, M., et al. (2010). Pre-vascularization of in vitro three-dimensional tissues created by cell sheet engineering. 31(14)**,** 3903-3909.

Chen, L., Xing, Q., Zhai, Q., Tahtinen, M., Zhou, F., Chen, L., et al. (2017). Pre-vascularization enhances therapeutic effects of human mesenchymal stem cell sheets in full thickness skin wound repair. 7(1)**,** 117.

Chen, W., Liu, X., Chen, Q., Bao, C., Zhao, L., Zhu, Z., et al. (2018). Angiogenic and osteogenic regeneration in rats via calcium phosphate scaffold and endothelial cell co‐culture with human bone marrow mesenchymal stem cells (MSCs), human umbilical cord MSCs, human induced pluripotent stem cell‐derived MSCs and human embryonic stem cell‐derived MSCs. 12(1)**,** 191-203.

Cipriano, J., Lakshmikanthan, A., Buckley, C., Mai, L., Patel, H., Pellegrini, M., et al. (2020). Characterization of a prevascularized biomimetic tissue engineered scaffold for bone regeneration. 108(4)**,** 1655-1668.

Dai, N.-T., Huang, W.-S., Chang, F.-W., Wei, L.-G., Huang, T.-C., Li, J.-K., et al. (2018). Development of a novel pre-vascularized three-dimensional skin substitute using blood plasma gel. 27(10)**,** 1535-1547.

Deegan, A.J., Hendrikson, W.J., El Haj, A.J., Rouwkema, J., and Yang, Y.J.b.j. (2019). Regulation of endothelial cell arrangements within hMSC–HUVEC co-cultured aggregates. 42(3)**,** 166-177.

Fan, Z., Liao, X., Tian, Y., and Nie, Y.J.A.b. (2020). A prevascularized nerve conduit based on a stem cell sheet effectively promotes the repair of transected spinal cord injury. 101**,** 304-313.

Fedorovich, N.E., Haverslag, R.T., Dhert, W.J., and Alblas, J.J.T.E.P.A. (2010). The role of endothelial progenitor cells in prevascularized bone tissue engineering: development of heterogeneous constructs. 16(7)**,** 2355-2367.

Freeman, F.E., Allen, A.B., Stevens, H.Y., Guldberg, R.E., McNamara, L.M.J.S.c.r., and therapy (2015). Effects of in vitro endochondral priming and pre-vascularisation of human MSC cellular aggregates in vivo. 6(1)**,** 1-18.

Freeman, F.E., Brennan, M.Á., Browe, D.C., Renaud, A., De Lima, J., Kelly, D.J., et al. (2020). A developmental engineering-based approach to bone repair: endochondral priming enhances vascularization and new bone formation in a critical size defect. 8**,** 230.

Fuchs, S., Hofmann, A., and Kirkpatrick, C.J.J.T.e. (2007). Microvessel-like structures from outgrowth endothelial cells from human peripheral blood in 2-dimensional and 3-dimensional co-cultures with osteoblastic lineage cells. 13(10)**,** 2577-2588.

Gholobova, D., Terrie, L., Mackova, K., Desender, L., Carpentier, G., Gerard, M., et al. (2020). Functional evaluation of prevascularization in one-stage versus two-stage tissue engineering approach of human bio-artificial muscle. 12(3)**,** 035021.

Heller, M., Frerick-Ochs, E., Bauer, H.-K., Schiegnitz, E., Flesch, D., Brieger, J., et al. (2016). Tissue engineered pre-vascularized buccal mucosa equivalents utilizing a primary triculture of epithelial cells, endothelial cells and fibroblasts. 77**,** 207-215.

Heo, D.N., Hospodiuk, M., and Ozbolat, I.T.J.A.b. (2019). Synergistic interplay between human MSCs and HUVECs in 3D spheroids laden in collagen/fibrin hydrogels for bone tissue engineering. 95**,** 348-356.

Hofmann, A., Ritz, U., Verrier, S., Eglin, D., Alini, M., Fuchs, S., et al. (2008). The effect of human osteoblasts on proliferation and neo-vessel formation of human umbilical vein endothelial cells in a long-term 3D co-culture on polyurethane scaffolds. 29(31)**,** 4217-4226.

Iqbal, F., Szaraz, P., Wu, J., Gauthier-Fisher, A., Li, R., and Librach, C.J.C. (2017). Co-administration of first trimester umbilical cord-derived perivascular cells (FTM HUCPVCs) with endothelial progenitor cells (EPCs) leads to enhanced angiogenesis, both in vitro and in vivo, compared to either cell type alone. 19(5)**,** S160-S161.

Jakubowska, W., Chabaud, S., Saba, I., Galbraith, T., Berthod, F., and Bolduc, S.J.T.E.P.A. (2020). Prevascularized Tissue-Engineered Human Vaginal Mucosa: In Vitro Optimization and In Vivo Validation. 26(13-14)**,** 811-822.

Jia, Z., Guo, H., Xie, H., Bao, X., Huang, Y., Yang, G., et al. (2018). Harvesting prevascularized smooth muscle cell sheets from common polystyrene culture dishes. 13(9)**,** e0204677.

Jiang, Y.N., Zhao, J., Chu, F.T., Jiang, Y.Y., and Tang, G.H.J.B.o. (2018). Tension-loaded bone marrow stromal cells potentiate the paracrine osteogenic signaling of co-cultured vascular endothelial cells. 7(6).

Kawecki, F., Clafshenkel, W.P., Auger, F.A., Bourget, J.-M., Fradette, J., and Devillard, R.J.B. (2018). Self-assembled human osseous cell sheets as living biopapers for the laser-assisted bioprinting of human endothelial cells. 10(3)**,** 035006.

Kérourédan, O., Hakobyan, D., Rémy, M., Ziane, S., Dusserre, N., Fricain, J.-C., et al. (2019). In situ prevascularization designed by laser-assisted bioprinting: Effect on bone regeneration. 11(4)**,** 045002.

Khan, S., Villalobos, M.A., Choron, R.L., Chang, S., Brown, S.A., Carpenter, J.P., et al. (2017). Fibroblast growth factor and vascular endothelial growth factor play a critical role in endotheliogenesis from human adipose-derived stem cells. 65(5)**,** 1483-1492.

Kim, B.-S., Chen, J., Weinstein, T., Noiri, E., and Goligorsky, M.S.J.J.o.t.A.S.o.N. (2002). VEGF expression in hypoxia and hyperglycemia: reciprocal effect on branching angiogenesis in epithelial-endothelial co-cultures. 13(8)**,** 2027-2036.

Kniebs, C., Kreimendahl, F., Köpf, M., Fischer, H., Jockenhoevel, S., and Thiebes, A.L.J.O. (2020). Influence of different cell types and sources on pre-vascularisation in fibrin and agarose–collagen gels. 16(1)**,** 14-26.

Kuss, M.A., Wu, S., Wang, Y., Untrauer, J.B., Li, W., Lim, J.Y., et al. (2018). Prevascularization of 3D printed bone scaffolds by bioactive hydrogels and cell co‐culture. 106(5)**,** 1788-1798.

Lee, J., Shin, D., Roh, J.L.J.H., and neck (2019). Promotion of skin wound healing using prevascularized oral mucosal cell sheet. 41(3)**,** 774-779.

Liu, H., Jiao, Y., Zhou, W., Bai, S., Feng, Z., Dong, Y., et al. (2018a). Endothelial progenitor cells improve the therapeutic effect of mesenchymal stem cell sheets on irradiated bone defect repair in a rat model. 16(1)**,** 1-13.

Liu, X., Chen, W., Zhang, C., Thein-Han, W., Hu, K., Reynolds, M.A., et al. (2017). Co-seeding human endothelial cells with human-induced pluripotent stem cell-derived mesenchymal stem cells on calcium phosphate scaffold enhances osteogenesis and vascularization in rats. 23(11-12)**,** 546-555.

Liu, X., Jakus, A.E., Kural, M., Qian, H., Engler, A., Ghaedi, M., et al. (2018b). Vascularization of natural and synthetic bone scaffolds. 27(8)**,** 1269-1280.

Lloyd-Griffith, C., McFadden, T.M., Duffy, G.P., Unger, R.E., Kirkpatrick, C.J., and O’Brien, F.J.J.A.b. (2015). The pre-vascularisation of a collagen-chondroitin sulphate scaffold using human amniotic fluid-derived stem cells to enhance and stabilise endothelial cell-mediated vessel formation. 26**,** 263-273.

Ma, J., Both, S.K., Ji, W., Yang, F., Prins, H.J., Helder, M.N., et al. (2014). Adipose tissue‐derived mesenchymal stem cells as monocultures or cocultures with human umbilical vein endothelial cells: Performance in vitro and in rat cranial defects. 102(4)**,** 1026-1036.

Manikowski, D., Andrée, B., Samper, E., Saint-Marc, C., Olmer, R., Vogt, P., et al. (2018). Human adipose tissue-derived stromal cells in combination with exogenous stimuli facilitate three-dimensional network formation of human endothelial cells derived from various sources. 106**,** 28-36.

Masuda, S., Matsuura, K., Anazawa, M., Iwamiya, T., Shimizu, T., and Okano, T.J.R.T. (2015). Formation of vascular network structures within cardiac cell sheets from mouse embryonic stem cells. 2**,** 6-16.

Miyazaki, H., Tsunoi, Y., Akagi, T., Sato, S., Akashi, M., and Saitoh, D.J.S.r. (2019). A novel strategy to engineer pre-vascularized 3-dimensional skin substitutes to achieve efficient, functional engraftment. 9(1)**,** 1-10.

Nguyen, B.N.B., Moriarty, R.A., Kamalitdinov, T., Etheridge, J.M., and Fisher, J.P.J.J.o.B.M.R.P.A. (2017). C ollagen hydrogel scaffold promotes mesenchymal stem cell and endothelial cell coculture for bone tissue engineering. 105(4)**,** 1123-1131.

Niknejad, H., Paeini-Vayghan, G., Tehrani, F., Khayat-Khoei, M., and Peirovi, H.J.P. (2013). Side dependent effects of the human amnion on angiogenesis. 34(4)**,** 340-345.

Ong, C.S., Fukunishi, T., Zhang, H., Huang, C.Y., Nashed, A., Blazeski, A., et al. (2017). Biomaterial-free three-dimensional bioprinting of cardiac tissue using human induced pluripotent stem cell derived cardiomyocytes. 7(1)**,** 1-11.

Pence, J.C., Clancy, K.B., Harley, B.A.J.B., and bioengineering (2015). The induction of pro‐angiogenic processes within a collagen scaffold via exogenous estradiol and endometrial epithelial cells. 112(10)**,** 2185-2194.

Peters, E.B., Christoforou, N., Leong, K.W., Truskey, G.A., West, J.L.J.C., and bioengineering, m. (2016). Poly (ethylene glycol) hydrogel scaffolds containing cell-adhesive and protease-sensitive peptides support microvessel formation by endothelial progenitor cells. 9(1)**,** 38-54.

Pirlo, R.K., Wu, P., Liu, J., Ringeisen, B.J.B., and bioengineering (2012). PLGA/hydrogel biopapers as a stackable substrate for printing HUVEC networks via BioLP™. 109(1)**,** 262-273.

Rambøl, M.H., Han, E., and Niklason, L.E.J.T.E.P.A. (2020). Microvessel network formation and interactions with pancreatic islets in three-dimensional chip cultures. 26(9-10)**,** 556-568.

Rosca, A.M., Mitroi, D.N., Cismasiu, V., Badea, R., Necula‐Petrareanu, G., Preda, M.B., et al. (2018). Collagen regulates the ability of endothelial progenitor cells to protect hypoxic myocardium through a mechanism involving miR‐377/VE‐PTP axis. 22(10)**,** 4700-4708.

Shimazu, Y., Zhang, B., Yue, Z., Wallace, G.G., Fukuda, J.J.J.o.b., and bioengineering (2019). Engineering of perfusable double-layered vascular structures using contraction of spheroid-embedded hydrogel and electrochemical cell detachment. 127(1)**,** 114-120.

Shor, E., Merdler, U., Brosh, I., Shoham, S., and Levenberg, S.J.B. (2018). Induced neuro-vascular interactions robustly enhance functional attributes of engineered neural implants. 180**,** 1-11.

Shudo, Y., Goldstone, A.B., Cohen, J.E., Patel, J.B., Hopkins, M.S., Steele, A.N., et al. (2017). Layered smooth muscle cell–endothelial progenitor cell sheets derived from the bone marrow augment postinfarction ventricular function. 154(3)**,** 955-963.

Skrzypek, K., Nibbelink, M.G., Karbaat, L.P., Karperien, M., van Apeldoorn, A., and Stamatialis, D.J.J.o.M.S.M.i.M. (2018). An important step towards a prevascularized islet macroencapsulation device—effect of micropatterned membranes on development of endothelial cell network. 29(7)**,** 1-15.

Sorrell, J.M., Baber, M.A., and Caplan, A.I.J.C.T.O. (2007). A self-assembled fibroblast-endothelial cell co-culture system that supports in vitro vasculogenesis by both human umbilical vein endothelial cells and human dermal microvascular endothelial cells. 186(3)**,** 157-168.

Valarmathi, M.T., Fuseler, J.W., Davis, J.M., Price, R.L.J.F.i.c., and biology, d. (2017). A novel human tissue-engineered 3-D functional vascularized cardiac muscle construct. 5**,** 2.

Xu, M., Li, J., Liu, X., Long, S., Shen, Y., Li, Q., et al. (2019). Fabrication of vascularized and scaffold-free bone tissue using endothelial and osteogenic cells differentiated from bone marrow derived mesenchymal stem cells. 61**,** 21-29.

Zhang, C., Hu, K., Liu, X., Reynolds, M.A., Bao, C., Wang, P., et al. (2017). Novel hiPSC-based tri-culture for pre-vascularization of calcium phosphate scaffold to enhance bone and vessel formation. 79**,** 296-304.

Zhang, W., Wray, L.S., Rnjak-Kovacina, J., Xu, L., Zou, D., Wang, S., et al. (2015). Vascularization of hollow channel-modified porous silk scaffolds with endothelial cells for tissue regeneration. 56**,** 68-77.

Zhu, S., Wang, J., Sun, Z.J.B., Biomimetic, and Nanobiomaterials (2018). Observation of co-culturing cells on porous silk fibroin films. 8(2)**,** 109-114.
